# Unbalanced 2 x 2 Factorial Designs and the Interaction Effect: A Troublesome Combination

**DOI:** 10.1371/journal.pone.0121412

**Published:** 2015-03-25

**Authors:** Johannes A. Landsheer, Godfried van den Wittenboer

**Affiliations:** 1 Department of Methodology and Statistics of Behavioral and Social Sciences, Utrecht University, Utrecht, The Netherlands; 2 Department of Education (Emeritus), University of Amsterdam, Amsterdam, The Netherlands; Fundacion Huesped, ARGENTINA

## Abstract

In this power study, ANOVAs of unbalanced and balanced 2 x 2 datasets are compared (N = 120). Datasets are created under the assumption that H1 of the effects is true. The effects are constructed in two ways, assuming: 1. contributions to the effects solely in the treatment groups; 2. contrasting contributions in treatment and control groups. The main question is whether the two ANOVA correction methods for imbalance (applying Sums of Squares Type II or III; SS II or SS III) offer satisfactory power in the presence of an interaction. Overall, SS II showed higher power, but results varied strongly. When compared to a balanced dataset, for some unbalanced datasets the rejection rate of H0 of main effects was undesirably higher. SS III showed consistently somewhat lower power. When the effects were constructed with equal contributions from control and treatment groups, the interaction could be re-estimated satisfactorily. When an interaction was present, SS III led consistently to somewhat lower rejection rates of H0 of main effects, compared to the rejection rates found in equivalent balanced datasets, while SS II produced strongly varying results. In data constructed with only effects in the treatment groups and no effects in the control groups, the H0 of moderate and strong interaction effects was often not rejected and SS II seemed applicable. Even then, SS III provided slightly better results when a true interaction was present. ANOVA allowed not always for a satisfactory re-estimation of the unique interaction effect. Yet, SS II worked better only when an interaction effect could be excluded, whereas SS III results were just marginally worse in that case. Overall, SS III provided consistently 1 to 5% lower rejection rates of H0 in comparison with analyses of balanced datasets, while results of SS II varied too widely for general application.

## Introduction

ANOVA is generally regarded as the best analysis techniques for balanced experiments that have equal number of subjects in each group: it is commonly held that it is both powerful and provides unbiased estimates. Some handbooks suggest that ANOVA also can be unbiased when unbalanced data are concerned, that is, when the condition of equal numbers of subjects for each treatment is not met. For example, a manual of SPSS (version 22) states: "ANOVA (analysis of variance) computes unbiased estimates using either the Type I or Type III sums of squares for each effect." [1]. Typically, sums of squares of Types II and III (SS II and SS III) are applied as correction methods when the data in an experiment are unbalanced. In a 2 x 2 factorial design, equal numbers in each group results in balance or orthogonality of the two factors and this ensures the validity of the comparison between the levels of the factors. The correction methods that have been developed for the case of unbalanced data, attempt to correct for non-orthogonal artifacts. They try to repair this with the intent to show how much of the effect of a treatment can be uniquely attributed to that treatment and does not partly result from the imbalance.

Imbalance occurs often in non-experimental and quasi-experimental designs for treatment research. Even when true experimental designs are balanced, an unequal number of subjects in each treatment condition can result from attrition or non-response. The core of the problem with unbalanced data is that in a factorial design the treatment contrasts become correlated or non-orthogonal when unequal numbers of subjects are present in the various groups. To some extent, this makes the estimates dependent on each other. Applying regular ANOVA, the correlated treatment contrasts result in variance components that are either too small or too large, dependent on the specific imbalance [2]. Therefore, the estimates of the main effects need correction, when the two effects of an unbalanced 2 x 2 design are analyzed in combination.

This problem has long been recognized [3] and procedures for the correction of this problem have been proposed. These correction procedures are known under several names and they primarily involve alternative ways to calculate the sums of squares (SS). In this paper, we use Type SS I as an identifier for the standard analysis that can be applied to balanced designs, and Type SS II and Type SS III as identifiers for the two ways to correct for imbalance (shortly indicated as respectively SS I, SS II and SS III).

The estimation of an interaction effect is often used in unbalanced designs as a criterion to decide between the two types of correction: when not statistically significant, it is considered negligible and SS II should be preferred. When the interaction is significant, SS III is the prevalent option. Another way to approach this decision would be to establish whether an interaction is theoretically viable or not. However, it is rare that researchers are entirely confident in the theoretical existence of such an interaction, especially in the case of research conducted in an area lacking strong theory.

Despite its long history [4] and its commonplace usage, ANOVA of unbalanced designs still leads to discussion and controversy [2,5,6]. Statistical software packages remain divided in their choice of defaults for ANOVA of unbalanced designs [2,6]. As in SPSS, the most popular choice among statistical packages is the use of SS III for correction of the analysis of unbalanced designs. In a 2 x 2 design, SS III adjusts for both variables and their interaction effect [6,7], while SS II adjusts each main effect for the other main effect, assuming no interaction effect. Although SS III has predominantly been used in the presence of an interaction, the unbiasedness of the resulting effect estimates has been questioned [5,6]: estimates based on SS III are considered to be too conservative. Both Langsrud and Macnaughton [5,6] argued, therefore, that SS II should always be the default to be applied, even in the presence of interaction. They stress that the use of SS II would result in more power to reject H0 of the main effects.

However, increased power has its limits. A rate of rejection of H0, after a correction for imbalance that is higher than the rate found in an equivalent balanced dataset (that is, of equal size and drawn from the same population) can be considered as undesirable, because the ANOVA of a balanced dataset needs no correction.

This leads to the following question: Does ANOVA offer sufficient power to reject H0 of the main effects when unbalanced designs are concerned? This will be examined with and without the presence of significant interactions. More specifically is the question whether the applications of the two correction methods lead to higher or lower rejection rates of H0 when compared to that of equivalent balanced datasets.

Not mentioned in the discussion thus far, however, is the additional problem that the estimation of interactions can be a problem in itself. It has been shown that when data are generated under H1 with specified non-zero effects, ANOVA does not necessarily allow these effects to be re-estimated. Landsheer, van den Wittenboer and Maassen [8] found that if only a multiplicative interaction effect between the two variables was operational in the generation of a balanced data set, the interaction effect remained often statistically insignificant while at the same time two significant main effects were found. This result is especially relevant for the analysis of unbalanced designs, as the estimation of the interaction plays a major role in the decision between SS II and III. Additionally, an unbiased estimate of the unique interaction effect has a high practical value. For instance, when two medicines strengthen each other’s effects, these medicines can be prescribed in lower dosages that may prevent or reduce side effects.

Since none of these aspects can easily be grasped in the framework of mathematical statistical theory, they are studied by means of simulation research. This study is a comparative power study, using simulated datasets in which the H1 of different effects is true. The main goal is to investigate whether SS II or SS III lead to higher or lower rejection rates of the main effects in the presence of different levels of interaction, in comparison to the results obtained for equivalent balanced datasets. A collection of 34 systematically varied unbalanced datasets has been used and, using the same simulation parameters, for each an equally sized balanced dataset is created. When using the same simulation parameters, we may assume that the balanced and unbalanced samples are drawn from the same population and that unbiased estimates will be similar, which will result in similar rejection rates of H0.

There is a variety of possibilities to create group differences and effects. Often, the method to create the data mimics the way they will be analyzed. We deviate from this rule, because in true research, it is only possible to make assumptions about the way effects are realized. One method for generating the data in 2 x 2 factorial designs under H1 assumes that there is no effect in the control group. The other method that we will use, assumes equal contributions to the results in both the treatment group and the control group. This latter method mimics the contrast coding that we will use when analyzing the data, while the first method does not. In addition, datasets are created both with and without interaction effects. Following earlier findings that interactions are not always re-estimated correctly [8], the question arises whether these different constructed datasets result in a satisfactory re-estimation of the interaction effect and how ANOVA of unbalanced datasets is influenced when the H0 of the interaction effect is rejected. We expect that ANOVA results are sensitive for different methods of effect construction. Specifically, the additional question is how a different construction of the effects influences the estimation of the interaction effect and subsequently the rejection rates of H0 of the main effects in the unbalanced datasets.

A detailed description of our simulation techniques, as well as details on the procedure used to evaluate the simulations, can be found in the Methods section. The Results section is devised in three subsections. In the subsection “Interactions in unbalanced samples”, we demonstrate the differences between SS I, II and III, the sizes of the corrections that are involved when applying SS II and III, and the influence that treatment or contrast coding has on these corrections. In the subsection “Transformation of the interaction effect”, we demonstrate the different effects of the two methods for data generation on the estimates of the interaction in balanced designs. In the subsection “Congruous and incongruous analysis of unbalanced datasets”, we present the influences of the two different data generation methods on the rejection rate of H0 of the interaction effects and the subsequent effect on the analysis of unbalanced datasets. In the congruous datasets the effects are created in a way that mimics how they are analyzed, while in the incongruous datasets the effects are created in a way that deviates from the applied analysis method. This last subsection is the comparative power study, in which the power obtained for main effects and interactions in simulated unbalanced datasets will be compared with the power obtained in equivalently simulated balanced datasets.

## Methods

### Venn diagrams

In ANOVA, the calculation of the sums of squares is central in the analysis of the data. ANOVA of a balanced 2 x 2 design produces unique SS components that can be attributed to the main effects, the interaction effect and the residual respectively. In unbalanced datasets, the total SS can be subdivided in a larger number of relevant parts. These extra components are non-unique and concern possible corrections of the standard components. They are not presented in regular analyses. These extra components concern parts of the total SS that are either added to or subtracted from the unique SS of the main effects. These extra components can be calculated with the use of different ANOVA analyses. When using SS I, the first entered variable is uncorrected, while the second is corrected for the first, the correctional component can be calculated by taking the difference of both estimates. We call these components non-unique or cross-predictive, as they can be part of two or more effects. Generally, ANOVA does not allow for entering an interaction effect as first predictor which is, nevertheless, needed for the calculation of some of the cross-predictive components. To circumvent this, the interaction effect has been calculated outside the ANOVA procedure and entered as an independent variable. This technique produced the same results as standard ANOVA, if the same order of factors has been used. The procedures in R [9] to calculate the various components are included in the supplemental files. When charting additive systems, Venn diagrams can provide an insightful representation [10,11]. In this paper, Venn diagrams are used to illustrate how the different additive SS components relate to each other and to indicate the corrections that are involved. Furthermore, these diagrams are helpful in understanding the differences between the types of SS.

### Coding systems for ANOVA

In ANOVA of a 2 x 2 design, the different treatment groups are coded with either treatment coding (0, 1) or with contrast coding (-1, 1). Clearly, these numbers do not represent the strength of the factors or causal agents but merely differentiate the treatments. In the statistical language R, coding with 0 and 1 is the default. This is identified as treatment coding, while the alternative coding procedure using-1 and 1 is named sum-to-zero contrasts in R. In this paper, we have used the term contrast coding for using-1 and 1. Both coding systems solve the over-parameterization problem within ANOVA [12] and imply specific restrictions. Also, the interpretation of the effects is slightly different: coding with 0 and 1 allows for direct interpretation of the main effects, while the interaction effect can be interpreted as the combined impact of both treatments over and above the impact of both main effects. The interaction effect in the fourth cell (where *A* * *B* = 1) is contrasted to the first three cells (where *A* * *B* = 0). As a result, the interaction effect is non-orthogonal to both factors, even in a fully balanced design. Therefore, the interaction effect is corrected for both *A* and *B* and the unique SS component is used. This correction is applied for all types of SS.

Coding with-1 and 1 enables interpretation of the differences of each condition from the grand mean. For the interaction effect, the use of-1 and 1 results in comparison of the diagonal cells 1 and 4 (A * B = 1) with the other two diagonal cells 2 and 3 (A * B = -1). The interpretation of the interaction effect is here that the impact of one treatment is dependent on the level of the other treatment, which is subtly different from the former interpretation. In balanced designs, the use of codes-1 and 1 makes the interaction effect orthogonal to both *A* and *B*.

### Simulation design

A basic design for comparative power studies has been followed. The statistics that have been tested are the F-tests of ANOVA in the 2 x 2 design, with the application of SS I for balanced datasets and both SS II and III for unbalanced datasets. The experimental data were simulated using a linear regression formula (Formula 1). *Y* represents the outcome; the two-level treatment factors *A* and *B* represent the factors or treatment conditions and *AB* the interaction between *A* and *B*; *β*_*0*_ is the intercept, *β*_*1*_, *β*_*2*_ and *β*_*3*_ represent both main effects and the interaction effect, respectively, and *ε* is the residual error:

$$Y=\beta_{0}+\beta_{1}A+\beta_{2}A+\beta_{3}AB+\varepsilon$$ (1)

The generated random samples have been specified by the alternative hypothesis (H1) for each of the effects for all of the conditions: *β*_*0*_ = 10, *β*_*1*_ = *β*_*2*_ = 4 and *β*_*3*_ for the interaction has been varied between 0, 4 and 8. The residual error *ε* was kept constant (10). The proportion of rejected null hypotheses out of 1000 simulations for each of the effects has been used to approximate the power of each test: each outcome is regarded as the outcome of a Bernoulli trial with a probability equal to the power. The “population” or true main effects in the simulations, expressed as betas, are moderate (4) in comparison to the error which were randomly drawn from a normal distribution with mean 0 and a standard deviation equal to 10. This resulted in a signal to noise ratio of. 4 for each main effect. Both causal factors *A* and *B* have an intermediate non-zero main effect in the population, and resulted therefore in H0 being rejected for most of the samples. With respect to the interaction effect, H0 is true when *β*_*3*_ = 0, while H1 is true for *β*_*3*_ = 4 and *β*_*3*_ = 8, covering an intermediate weak and a strong interaction effect, respectively.

Commonly, the different statistical approaches to be compared are applied to the same simulated datasets and the results show unequivocally which approach is the most powerful. But when comparing unbalanced with balanced data, the datasets themselves are different. An unbalanced sample (for example, 40, 20, 30, 30) in the four cells of a 2 x 2 experimental design differs always from a balanced sample (for example, 30, 30, 30, 30), because different numbers of subjects are treated differently. This hinders a direct comparison of the power of the various methods that can be applied to unbalanced datasets with those applied to balanced data. Therefore, an extended approach has been used: a systematically constructed set of unbalanced datasets has been created, which allowed us to study the impact of different features of the unbalanced designs on the rejection rates of the three null hypotheses.

The set of unbalanced datasets was constructed as follows. It was desirable that an unbalanced dataset had a balanced counterpart of the same size. We started with the smallest balanced 2 x 2 design that allowed for conversion into unbalanced designs of the same size, such that variance would exist in each cell of the design. This implied that each unbalanced design had to have at least a frequency of 2 in each cell. Consequently, size 8 with frequencies (2, 2, 2, 2) is too small, since conversion to an unbalanced design would always lead to a cell with one subject and, therefore, no variance. A size of 12 with frequencies (3, 3, 3, 3) satisfied the restrictions. The number of ways to split *n* indistinguishable objects into *k* distinct categories is *C* (*n* + *r*—1, *k*—1). For a 2 x 2 design, 12 randomly selected subjects can be divided over 4 groups in 455 different ways. These also include cells with only one or zero subjects and these divisions over the cells were deleted, resulting in one balanced dataset over the four cells and a systematic variety of 34 unbalanced datasets. The code for doing this can be found in S2 File. The unbalanced datasets are all draws of four samples out of 12 subjects with a minimum of two subjects for each of the four cells. Because the use of only 12 subjects proved to result in too large sampling errors, it was decided to use a multiple of 10 for each cell, resulting in 120 simulated subjects in each analysis. Of course, 120 subjects could have been divided in considerably more unbalanced datasets than 34. However, the variation of 34 unbalanced datasets was sufficient for our purposes: it resulted in a systematic variation of the unbalanced datasets over a broad range of possibilities and makes it promising to study the influence of imbalance itself on the obtained effect estimates. Each cell of the dataset contained 60, 50, 40, 30 or 20 subjects. The 34 unbalanced datasets are presented in Table 1. The largest imbalance is threefold (60 versus 20), while the balanced design has an equal number of subjects in all cells (30, 30, 30, 30).

**Table 1:** Systematic variations of 34 unbalanced 2 x 2 designs with 120 subjects.

	Cells					Cells					Cells					Cells			
Nr	00	01	10	11	Nr	00	01	10	11	Nr	00	01	10	11	Nr	00	01	10	11
**1**	20	20	20	60	**10**	20	40	20	40	**19**	30	20	50	20	**27**	40	20	40	20
**2**	20	20	30	50	**11**	20	40	30	30	**20**	30	30	20	40	**28**	40	30	20	30
**3**	20	20	40	40	**12**	20	40	40	20	**21**	30	30	40	20	**29**	40	30	30	20
**4**	20	20	50	30	**13**	20	50	20	30	**22**	30	40	20	30	**30**	40	40	20	20
**5**	20	20	60	20	**14**	20	50	30	20	**23**	30	40	30	20	**31**	50	20	20	30
**6**	20	30	20	50	**15**	20	60	20	20	**24**	30	50	20	20	**32**	50	20	30	20
**7**	20	30	30	40	**16**	30	20	20	50	**25**	40	20	20	40	**33**	50	30	20	20
**8**	20	30	40	30	**17**	30	20	30	40	**26**	40	20	30	30	**34**	60	20	20	20
**9**	20	30	50	20	**18**	30	20	40	30										

For each of the 34 unbalanced datasets, each of the three interaction effect conditions and each of the two effect construction methods, 1000 samples were drawn. For each simulation, a sample was drawn that was large enough to fill both the unbalanced and the balanced datasets. For each unbalanced dataset, the same simulated subjects were used as much as possible for the equivalent balanced dataset and each subject in each group was simulated with the same parameters. Assuming unbiased estimates, the mean results of 1000 simulations should provide in principle about the same estimates for each tested dataset and the same rejection rates. Supplemental files with the R code of the various analysis procedures are available.

### Generating effects

We have used two different methods to create the effects for 2 * 2 designs under H1. The first method is called *generation of zero-effect control data* and uses the values (0, 1) for *A* and *B* in Formula 1. Applying this method of data generation assumes that the control groups of both *A* and *B* have no effect (similar to no-treatment control), while each treatment condition does have an effect and both treatments in combination may have a multiplicative interaction effect of three different strengths. Therefore, a true interaction resulted in a zero contribution to the results in the first three cells ((0, 0), (0, 1) and (1, 0)) and in a non-zero contribution to the results in the fourth cell (1, 1).

The second method of generating effects is called *generation of equal-contribution data* and uses (-1, 1) for *A* and *B* in Formula 1. This data generation method assumes that both control groups and treatment groups of the factors *A* and *B* show equal but contrasting contributions to the group differences, while the interaction of both factors results in a contrast of the cells (-1, -1) and (1, 1) with the other two cells (-1, 1) and (1, -1).

The generation of “zero-effect control” data seems the natural companion of analysis using treatment coding, with 0 representing no effect, while the generation of “equal contribution” data seems to be more in concordance with analysis using contrast coding. Please note that the data generated with the two different methods of effect construction are quite dissimilar, most specifically for the interaction effect. Furthermore, although the creation of data looks like the use of a coding system for analysis, it has little to do with it. In true research, the ways effects are realized is a hidden process and it is only possible to formulate assumptions about them. However, the impact of the different ways effects are realized can be studied using simulations.

### Indicator of unbalance

For additional interpretation of some of the results, an indicator of unbalance has been used. The unbalance *U* can be expressed for each effect *V* as:

$$U_{V}=\frac{f_{V=T}-f_{V=C}}{N}$$ (2)

The term in the numerator is the contrast in numbers for each effect, where *V* represents the effect (either main effect *A*, *B* or interaction *AB*) and *f*_*V = x*_ is the sum of the cell counts if *V* identifies treatment (*T*) or control (*C*).

## Results

### Interactions in unbalanced samples

**Fig 1:**
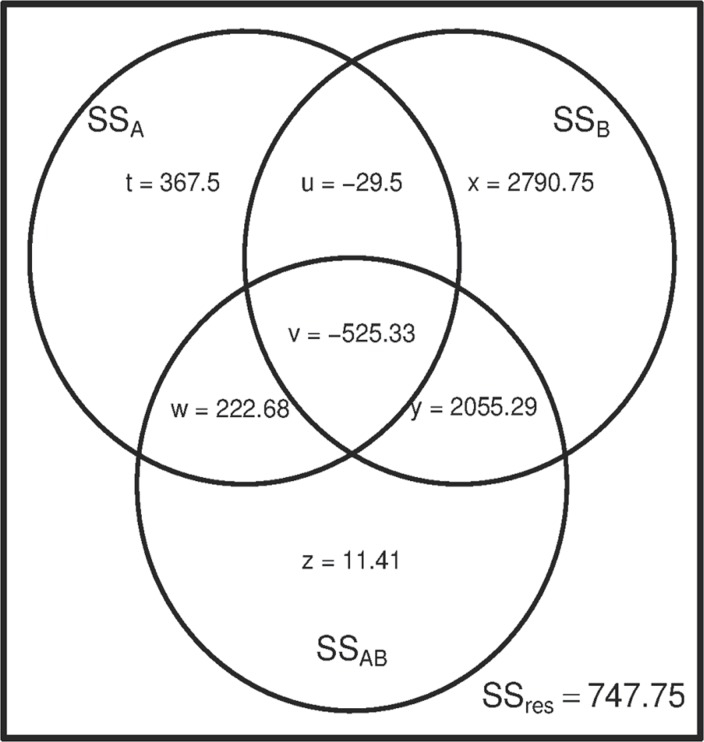
Venn diagram of the example data [13], using treatment coding (0, 1).

In this section, we demonstrate the features of the different sums of squares. Table 2 and Fig. 1 shows all SS components of an unbalanced sample [13] when using treatment coding (0, 1) for ANOVA. The dataset is provided in the supporting information file S8 File”, which also contains the code to construct the tables and figures of this section.

**Table 2:** The SS of example data, using (0, 1) coding for analysis.

	SS IA		SS IB		SS II		SS III	
Model	*Y ~ A + B + AB + e*		*Y ~ B + A + AB + e*		*Y ~ A + B + AB + e*		*Y ~ A + B + AB + e*	
	SS	components	SS	Components	SS	components	SS	components
***A***	35.3	t + u + v + w	590.2	t + w	590.2	t + w	367.5	t
***B***	4846.0	x + y	4291.2	u + v + x + y	4846.0	x + y	2790.8	x
***A*:*B***	11.4	z	11.4	z	11.4	z	11.4	z
**Residuals**	747.8		747.7		747.8		747.8	

In Table 2 and Fig. 1, the components *t*, *x* and *z* represent the unique components of variable *A*, *B* and the interaction effect *AB* (the SS III components), while *u*, *v*, *w* and *y* represent the non-unique or cross-predictive components. It should be noted that the use of (0, 1) coding for analysis with SS III is not recommended. The SS I estimates are dependent on the order in which the two factors are entered.

The SS II components for *A* and *B* are respectively *t + w* and *x + y*. As the interaction effect is assumed negligible, SS II for the interaction effect is not well defined, but all statistical packages attribute only unique part *z* to the interaction effect. For the model of Y, *Y ~ A + B + AB + e*, the SS IA components are *t + u + v +w* (variable *A*), *x + y* (variable *B*) and *z* (interaction effect *AB*). For the differently ordered model *Y ~ B + A + AB + e*, the SS IB components are *x + y + u + v* (variable *B*), *t + w* (variable *A*), and *z* (interaction effect *AB*). The total explained variance is the sum of all components: *t + u + v + w + x + y + z* and the total variance of the dependent variable *Y* is the total of explained variance plus the SS of the residuals (SS_*res*_). In this example, it is easy to see that the non-unique components *u*, *v*, *w* and *y* can be large, absolutely as well as relatively.

More importantly, we see that the components *u* and *v* are negative. For correction, these cross-predictive components *u* and *v* are attributed to *both A and B* when using SS II, as result of subtracting a negative quantity. Because of such negative components the sum of the unique parts *t*, *x* and *z* can be larger than the total explained variance and even larger than the total of variance of the dependent variable. This is the reason that SS II and III are called non-additive. They are only additive if all variance components are considered with the inclusion of all non-unique components (Fig. 1). The negative, non-unique SS components are seemingly in error, as sums of squares are inherently positive. In similar cases, Nelder concluded that “The appropriate formulae should be applied as though the component were negative and no special distinction should be made”(p. 548) [14]. The relevant question is whether and when the non-unique components provide a sensible correction for the main effects.

An incorrect formulation would be that the correction in SS II and III concerns overlapping variance and that it reduces overestimation of the main effects. In fact, the correction can concern both overlapping and underlapping sums of squares [2]. In the case of underlapping, the amount of variance that is used for correction is negative, and it will be assigned to both variables; the correction possibly diminishes underestimation. We prefer the terms cross-predictive or non-unique instead of “overlapping”, because it represents the influence of one factor on the other.

The application of SS I, II and III will lead to identical results for balanced designs. The resulting dataset of a successfully executed balanced 2 x 2 factorial design is orthogonal with respect to the two factors *A* and *B*, but not necessarily to their interaction effect *AB*, because this depends on the coding of the two factors during the analysis. In the analysis, the interaction is orthogonal to the main effects if the codes-1 and 1 are used and non-orthogonal if coded 0 and 1.

**Fig 2:**
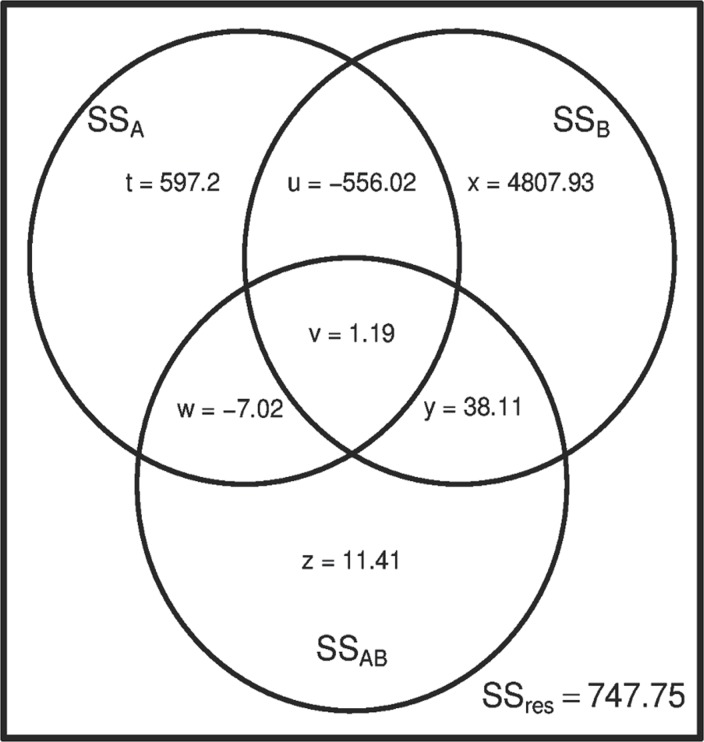
Venn diagram of the example data [13], using contrast coding (-1, 1).

Table 3 and Fig. 2 show the results when contrast coding (-1, 1) is used for the analysis. For the unbalanced datasets, the use of codes-1, 1 makes a large difference (Fig. 2): the cross-predictive components *v*, *w* and *y* are much smaller and *v* and *w* have changed their sign. As a result, the unique components (SS III) of *A* and *B* have increased in comparison to the estimates shown in Fig. 1. Please note that the sum of *u* + *v* is equal in Figs. 1 and 2. SS II provides equal estimates for the main effects (*t +w* and *x + y*, respectively) independent of the codes used, and can differ considerably from SS III estimates; SS III estimates differ for the two coding systems. Please note that for the analysis of a given sample, the use of the two different coding systems leads to the same results, with the exception of SS III. When using SS III, only the unique parts are considered for effect estimation. In general, the use of (-1, 1) coding is to be recommended in that case.

**Table 3:** The SS of Example data, using (-1, 1) coding for analysis.

	SS IA		SS IB		SS II		SS III	
Model	*Y ~ A + B + AB + e*		*Y ~ B + A + AB + e*		*Y ~ A + B + AB + e*		*Y ~ A + B + AB + e*	
***A***	35.3	t + u + v + w	590.2	t + w	590.2	t + w	597.2	t
***B***	4846.0	x + y	4291.2	u + v + x + y	4846.0	x + y	4807.9	x
***A*:*B***	11.4	z	11.4	z	11.4	z	11.4	z
**Residuals**	747.7		747.7		747.8		747.8	

The Venn diagrams illustrate that the corrections made when using SS II and III can be considerable. Furthermore, the corrections are sometimes negative and result in larger estimates for the SS that is attributed to the main factor. With respect to the coding system used in ANOVA of an unbalanced dataset, our results show that contrast coding leads to smaller non-unique components than treatment coding.

### Transformation of the interaction effect

In this subsection, we show the influence of differently constructed effects on the estimate of the interaction effect. When analyzing 2 x 2 datasets in real life, there is no certain way to know with which exact parameters the data are realized. Using simulations, however, we can generate our own data and show whether the parameters of the data generation are re-estimated correctly and when this is not the case. To illustrate this for ANOVA, we use a single large dataset of 1000 subjects in a balanced 2 x 2 design for which in principle the parameters can be re-estimated without too much deviation. The betas used for the data generation are equal for both main effects and interaction, respectively: *β*_1_ = *β*_2_ = *β*_3_ = 4, with a random error of 10. Of course, the effects are all highly significant in this large sample, however in our case it is only the SS that is of interest. Our expectation should be that the two main effects and the interaction effect have equal *SS*, as they are generated with equal betas. For the data generation, the two different methods discussed in the Methods section have been used: the “no-effect control” data generation method and the “equal-contribution” data generation method. Both resulting datasets are quite different, but we focus here on the differences between the two coding systems in ANOVA within each type of data generation.

Table 4 presents the results of a balanced dataset, both for re-estimation of betas and SS. The re-estimation of the interaction effect is supposed to be equal to the main effects; Table 4 shows the results of 1000 simulations.

**Table 4:** Congruous and incongruous data analysis.

Effect construction		(0,1)					(-1, 1)				
Analysis			(0, 1)		(-1, 1)			(0, 1)		(-1, 1)	
Effect	df	*β*	*β^*	SS	*β^*	SS	*β*	*β^*	SS	*β^*	SS
**Intercept**		10	10.0		15.0		10	6.0		10.0	
***A***	1	4	3.9	9000	3.0	9000	4	-.1	15966	4.0	15966
***B***	1	4	4.0	9170	3.0	9170	4	0.0	16191	4.0	16191
***AB***	1	4	4.1	1141	1.0	1141	4	16.1	16276	4.0	16276
**Residual**	996	10	10.0	99786	10.0	99786	10	10.0	99786	10.0	99786

***β*** true beta; ***β*** ^*^*^ estimate

Examining the re-estimated betas (***β*** ^***^***^), we see that data analysis with linear regression leads to precise re-estimation of the betas, provided that the effect construction in the generated data and the coding for analysis is congruous. In that case, the data generation mimics the coding used in the analysis method. Incongruous analysis, in which the coding system used in the linear regression is not mimicked, shows highly divergent results. For ANOVA, however, we see a different pattern: when data are generated with (0, 1), the SS of the interaction effect *AB* is but a fraction of the SS of either main effect. Furthermore, there is no difference in using either coding system in the analysis of this balanced dataset. When the data are generated with (-1, 1), the interaction effect is about the same as both main effects, independent of the coding used for analysis.

Examining the means can help to understand the differences in the ANOVA analyses. The effect construction with the assumption of no effect in the control conditions and using (0, 1) does exactly what it has to do, but it has a side effect on the ANOVA estimates when an interaction effect in the form of a multiplication occurs (and most interaction effects imply some form of multiplication). Let us enumerate the four cells from left-to-right and from top-to-bottom. Effect construction using (0, 1) with three betas of 4 for the main effects and their interaction, contributes (neglecting the intercept of 10) zero to the outcome in cell 1, four to cells 2 and 3 and twelve to cell 4, resulting in a grand total of twenty. The expected differences for the group that received the treatment of factor *A*, and the group that received the control condition of factor *A* is therefore 8–2 = 6 (the mean of 12 and 4, minus the mean of 0 and 4), and similarly for the difference between the group that received treatment *B* and the control group of *B*. Without interaction, the group differences would have been 4 between both conditions. In other words, the main effects have been increased because of the interaction. Moreover, without interaction the total mean of 4 (still neglecting the intercept) increases to 5 when the interaction is added. Using (0, 1) coding the interaction effect increases the main effects and the total mean for a great deal, while ANOVA only attributes the remainder to the interaction. As a result, the interaction estimate is smaller than the true contribution when both treatments are combined, while both main effects are larger. The interaction can be found insignificant in small samples. When the effect is constructed with (-1, 1), with both control groups and treatment groups contributing to the effect, the additional contribution of the interaction effect that occurs in cells 1 and 4 is leveled off in the cells 2 and 3. Therefore, the contribution of the interaction in the (-1, 1) method will only benefit the interaction effect to be estimated.

Both for balanced datasets (Table 4) and unbalanced datasets (compare Table 2 and 3), the two coding systems in ANOVA result in the same effect estimates for the main factors and their interaction when applying SS I or II. In the following sections, we will use only contrast coding for analysis, because this reduces the corrections necessary for unbalanced datasets and is a necessity when using SS III. Clearly, underestimation of the interaction effect can be found in balanced datasets. The specific importance of this phenomenon for unbalanced datasets is that a lower estimate of the real interaction effect leads more often to the choice of SS II, as the interaction will be statistically insignificant more often. In the next section, we will delve more deeply into the impact of unbalanced datasets with differently constructed effects on the rejection rates of H0.

### Unbalanced congruous and incongruous data analysis

#### Congruous data analysis

In this subsection, we present the results of congruous simulations and data analyses, when all effects are both generated and analyzed with (-1, 1) coding. In these datasets the effects are constructed with an equal contribution of both the control and the treatment group to the effect of each factor. The overall influences of different interaction effects on the balanced and 34 unbalanced datasets are shown in Table 5. This table shows the mean rejection rates of H0 over all simulations and all analyzed samples. Each row contains the means of 34000 simulations: 1000 simulations for each of the 34 unbalanced sample datasets. In each row all datasets are created with the same parameters, that is, we simulate that they are drawn from the same population in which the H1 for the main effects is true. The strength of the interaction effect is varied from 0 (H0 is true) to 4 and 8 (H1 is true). For each of the 34 datasets, unbiased estimates over 1000 simulations should therefore be similar and result in similar rejection rates of H0.

**Table 5:** Mean rejection rates of H0 (congruous data analysis).

	Balanced			Unbalanced, SS II			Unbalanced, SS III		
Beta of interaction	*A*	*B*	*AB*	*A*	*B*	*AB*	*A*	*B*	*AB*
**0**	0.586	0.583	0.051	0.549	0.549	0.050	0.535	0.536	0.050
**4**	0.586	0.577	0.587	0.543	0.542	0.537	0.536	0.533	0.537
**8**	0.586	0.584	0.992	0.537	0.538	0.982	0.535	0.534	0.982

In Table 5, we see a stable result. When balanced datasets are used, the H0 of both *A* and *B* are rejected in about 59% of the simulations. For the unbalanced datasets, the rejection rates are somewhat lower if SS II is used, about 55%. It is even lower if SS III is used, about 53.5%. When SS II is used, the rejection rate of both main effects depends slightly on the true value of the interaction effect. The rejection rates of SS III for both main effects are almost equal, independent of the value of the interaction effect in the population. Estimating the interaction effect *AB*, the mean rejection rates of H0 are independent of whether SS II or III has been used. Of course, the rejection rates increase, as the interaction effect becomes stronger.

Table 6 presents the number of unbalanced datasets that result in a higher or lower rejection rate over 1000 simulations in comparison with the rejection rate of the balanced dataset for the three different sizes of the interaction.

**Table 6:** Number of unbalanced designs (percentages of all 34 designs) with rejection rates of H0 that are higher or lower when compared to the rejection rates of the balanced data (congruous data).

			Unbalanced, SS II			Unbalanced, SS III		
Beta of interaction	Rejection rate		*A*	*B*	*AB*	*A*	*B*	*AB*
**0**	Higher	# (%)	1 (2.9%)	3 (8.8%)	16 (47.1%)	0 (0%)	0 (0%)	16 (47.1%)
	Δ	0.009	0.012	0.006			0.006
	Lower	# (%)	32 (94.1%)	31 (91.2%)	14 (41.2%)	34 (100%)	34 (100%)	14 (41.2%)
			Δ	-0.040	-0.039	-0.008	-0.051	-0.047	-0.008
**4**	Higher	# (%)	14 (41.2%)	15 (44.1%)	0 (0%)	0 (0%)	0 (0%)	0 (0%)
		Δ	0.099	0.099				
	Lower	# (%)	20 (58.8%)	19 (55.9%)	34 (100%)	34 (100%)	34 (100%)	34 (100%)
			Δ	-0.142	-0.143	-0.050	-0.049	-0.044	-0.050
**8**	Higher	# (%)	15 (44.1%)	15 (44.1%)	1 (2.9%)	0 (0%)	0 (0%)	1 (2.9%)
		Δ	0.192	0.197	0.004			0.004
	Lower	# (%)	19 (55.8%)	19 (55.8%)	32 (94.1%)	34 (100%)	34 (100%)	32 (94.1%)
			Δ	-0.239	-0.237	-0.010	-0.050	-0.050	-0.010

# (%) number of designs (percentage); Δ mean difference in rejection rates of H0

Table 6 shows that when beta is zero and the H0 of the interaction is true, SS II produces slightly higher rejection rates of H0 of the main effects in a few of the datasets, when compared to a balanced dataset. In the majority of datasets SS II results in a lower rejection rate. SS III is a bit more conservative as it rejects H0 of the main effects less often, while the differences in the rejection rates (Δ) are about. 05. The differences in the higher rejection rates become more considerable for SS II when beta 0 and beta 4 are being compared and even higher for beta 8. If the strength of the interaction effect is 4, the rejection rate is about 10% higher when using SS II than in the balanced analysis. It is about 20% higher if the strength of the interaction effect is 8. These higher rejection rates are found in more than 40% of the unbalanced designs. Looking at the lower rejections rates, the difference in comparison with the balanced design (Δ) increases when the size of the interaction increases from 0 to 8. When an interaction is present, SS II produces either higher or lower estimates, resulting in higher or lower rejection rates of H0 of the main effects. The variation is considerable. This results in a higher rejection of H0 of the main effects in presence of an interaction effect for more than 40% of the designs. Most of the problematic designs can be identified. The imbalance in each dataset can be quantified using Formula 2, and an imbalance of one factor larger than zero indicates a higher rate of rejection of the other factor.

In contrast, when using SS III, there is no higher rejection of H0: in every design, there is a lower rejection rate of H0 of the main effects and this difference is consistently about 5% when compared to the rejection of the comparable balanced design.

#### Incongruous data analysis

We now present the results for the incongruous method of data generation and analysis. The effects in the data are generated with (0, 1), using the assumption that no effect is found in a no-treatment control group and that group differences are solely the result of the treatments. The overall impact of different interaction effects on the balanced dataset and 34 unbalanced datasets are shown in Table 7. This table is constructed in the same way as Table 5, but analyses using treatment coding are now applied to datasets in which the effects are constructed in a way that does not mimic the method of analysis.

**Table 7:** Mean rejection rates of H0 (incongruous analysis).

	Balanced			Unbalanced, SS II			Unbalanced, SS III		
Beta of Interaction	*A*	*B*	*AB*	*A*	*B*	*AB*	*A*	*B*	*AB*
**0**	0.586	0.583	0.051	0.549	0.549	0.050	0.535	0.536	0.050
**4**	0.903	0.902	0.197	0.874	0.871	0.180	0.867	0.865	0.180
**8**	0.991	0.991	0.579	0.982	0.980	0.529	0.984	0.983	0.529

Table 7 shows that the multiplicative interaction effects are partly transformed into main effects. When a true interaction effect that is as strong as the main effects is present (beta = 4), unexpectedly low rejection rates of H0 for the interaction effect have been found, while the rejection rates of the main effects have become considerably higher. Consequently, the main effects are considerably overestimated, all the more so if the value of the simulation parameter beta that represents the true interaction effect is higher. As the H0 of the interaction effect is accepted in more cases, S II seems more often the suitable choice for correction.

As can be seen in Table 7, the rejection rates of H0 of the main effects strongly depend on the strength of the interaction effect: the rejection rates of both the main effects increase considerably when the true interaction is larger. This dependency can be observed in both the balanced and the unbalanced datasets using SS II and SS III. When the beta of the interaction is zero and four, SS III is a little more conservative, while the differences between SS II and SS III are small when beta is eight.

Table 7 does not present the complete story, however. Like Table 6, Table 8 presents the number of unbalanced datasets that result in higher or lower rejection rates over 1000 simulations in comparison with the rejection rate of the corresponding balanced dataset.

**Table 8:** Number of unbalanced designs (percentages of all 34 designs) with rejection rates of H0 that are higher or lower when compared to the rejection rates of the balanced data (incongruous data).

			Unbalanced, SS II			Unbalanced, SS III		
Beta of interaction	Rejection rate		*A*	*B*	*AB*	*A*	*B*	*AB*
**0**	Higher	# (%)	1 (2.9%)	3 (8.8%)	16 (47.1%)	0 (0%)	0 (0%)	16 (47.1%)
		Δ	0.009	0.012	0.006			0.006
	Lower	# (%)	32 (94.1%)	31 (91.2%)	14 (41.2%)	34 (100%)	34 (100%)	14 (41.2%)
		Δ	-0.040	-0.039	-0.008	-0.051	-0.047	-0.008
**4**	Higher	# (%)	8 (23.5%)	7 (20.6%)	2 (5.8%)	0 (0%)	0 (0%)	2 (5.9%)
		Δ	0.032	0.029	0.011			0.011
	Lower	# (%)	26 (76.5%)	19 (55.9%)	32 (94.1%)	33 (97.1%)	34 (100%)	32 (94.1%)
		Δ	-0.047	-0.048	-0.019	-0.036	-0.037	-0.019
**8**	Higher	# (%)	8 (23.6%)	7 (20.6%)	0 (0%)	0 (0%)	0 (0%)	0 (0%)
		Δ	0.006	0.006				
	Lower	# (%)	23 (67.6%)	23 (67.6%)	34 (100%)	33 (97.1%)	32 (94.1%)	34 (100%)
		Δ	-0.016	-0.018	-0.050	-0.008	-0.009	-0.050

# (%) number of designs (percentage); Δ mean difference in rejection rates of H0

When the true interaction effect is zero (beta = 0) and when applying SS II, Table 8 shows in a few of the designs a slight overestimation of the main effects and a higher rejection rate of H0 of both main effects. Both the number of unbalanced designs involved and their differences in rejection rates are limited. SS III provides more often lower rejection rates in comparison to the results for the balanced dataset, with a difference of about 5%.

If the true beta of the interaction effect is 4, the comparison with the rejection rates of the balanced datasets becomes worse for SS II. The number of unbalanced designs that cause a higher rejection rate of H0 of both main effects increases to about one in five, while the difference (Δ) of the rejection rates increases to about 3%. In a far larger number of the designs, a lower rejection rate of the main effects can be found, resulting in almost 5% less rejections. Interestingly enough, the problematics designs are no longer identifiable: in contrast to the congruous analysis, the unbalance itself is no longer indicative of higher or lower rejection rates. When applying SS III, the rejection rates of the main effects are never higher and always lower in comparison to the equivalent balanced datasets. When the rejection rates are lower, the Δ is about 1% better when SS III is applied instead of SS II, while higher rejection rates do not occur when using SS III.

A further increase of the interaction (beta = 8), does not result in a further increase in the amount of main effects H0 rejections in the unbalanced datasets when SS II is applied. Instead, rejection rate differences decrease for SS II in comparison with the results obtained for the smaller interaction (beta = 4) and are almost equal to those obtained for the balanced design. Furthermore, the differences between SS II and SS III are small, though slightly in favor of SS III, with SS III providing exclusively lower rejection rates of H0, and in no case higher rejection rates.

The results look better for SS II in this table, when compared with the results of SS II in Table 6. Higher rejection rates occur in a smaller number of designs, and the differences (Δ) are smaller. The results for the interaction effects are similar when using SS I, II or III. In these types of incongruous analyses, significant estimates of interaction effects occur more rarely, and in Table 7 we can see that the interaction effect is not statistically significant in about 47% in the unbalanced datasets, even when the true beta of the interaction is 8. However, in comparison to the rejection rates of the balanced dataset, application of SS III leads systematically to a lower or equal rejection rate of H0 of the main effects and the differences Δ are smaller.

## Discussion

Our main conclusion is that ANOVA with SS II only works satisfactorily for the unbalanced designs when an interaction can be excluded, while applying SS III when an interaction is present showed a power of about 1 to 5% less than for the balanced datasets that were simulated with the same parameters. When applying SS II in that case, the power of ANOVA for the unbalanced datasets varied from 20% higher power to 24% lower power. This variation is undesirable as well as the risk of an inflated Type I Error. The application of SS II cannot be recommended when an interaction is present in an unbalanced design. However, a part of our results seems to be in support of Langsrud’s claim [5] that SS II leads to a higher power in comparison to SS III and is therefore preferable, even in the presence of an interaction. The details of the results taught otherwise.

We have studied two different methods to create the effects in the simulated datasets, assuming that H1 is true. If *balanced* datasets are generated randomly under H1, with the additional assumption that the control conditions offer no contribution to the main effects and the interaction, our results indicate that the interpretation of the unique effects that are estimated by ANOVA is problematic. The interaction effect is underestimated and its H0 is rejected far less frequently than it should be, while the main effects are overestimated and the rejection rates of their H0 become considerably higher. A similar phenomenon has been demonstrated in earlier research [8]. The *complete* ANOVA models still provide reasonable estimates for individual cases and the various effects can be interpreted *in combination with each other*. It is the interpretation of the separate effects that is problematic. Because the estimate of the interaction is often used for the choice of the correction method that should be applied to unbalance data, this finding has special relevance for unbalanced data.

When unbalanced datasets are simulated randomly under H1, with the additional assumption that both control groups and treatment groups contribute equally to the main effects and the interaction, the correction procedures SS II and SS III both seem to work fine. Combining the results of all 34 unbalanced datasets, both correction methods provide conservative estimates. These overall results showed a higher power for SS II than for SS III, which seemingly corroborates Langsrud’s viewpoint. In these simulated datasets, the unique interaction is re-estimated correctly. SS II offers the best correction for data with a negligible interaction. In the presence of an interaction SS II resulted in estimates that had a high variation. In these datasets, an imbalance in one factor causes overestimation of the effect of the other factor when applying SS II in the presence of an interaction. This increases when the interaction is larger. Dependent on the specific imbalance found in the dataset, using SS II can lead to rejection rates of H0 that are higher than those obtained from the equivalent balanced dataset. We consider this as undesirable. Alternatively, SS III results solely in lower rejection rates of H0 of the main effects in the presence of an interaction. These results show 4 to 5% more rejections of H0 of the main effects when compared to the rejection rates found in equivalent balanced samples. In these datasets, the application of SS III offers the better correction method in the presence of an interaction, because the results of SS II vary too widely. When applying SS II, the higher rejection rates that we found in some of the unbalanced designs are considerable and unacceptably high in comparison to an alpha of. 05.

The picture changes considerably when unbalanced datasets are simulated randomly under H1, with the additional assumption that only treatment groups contribute to the main effects and the interaction, while the control groups show no effect. This situation may be expected in experiments with no-treatment control groups. In these simulated datasets, the unique interaction effect is *not* re-estimated correctly. Unbalanced 2 x 2 factorial designs and interaction effects are a troublesome combination in this case. In these datasets, SS II seems to be applicable far more often, as the H0 of the interaction effect is not rejected frequently. The correction of SS II seems to work better on these datasets, even in the presence of a true interaction. In comparison with the analysis of balanced datasets, higher estimates are found more rarely and the size of the rejection rate differences is smaller for the main effects. Again, these results seem to corroborate Langsrud’s viewpoint. However, SS III performs better. Firstly, it never results in undesired higher rejection rates of both main effects when compared to the analysis of balanced datasets, while SS II still shows higher rejection rates with these kinds of simulated datasets. Secondly, the amount of lower rejection rates in comparison with ANOVA applied to balanced datasets from the same population is slightly better than what SS II provides. The seemingly improved functioning of the correction with SS II can be understood in the light of the fact that the interaction is also underestimated in the balanced datasets. Interestingly enough, the imbalance in the design is no longer useful in predicting SS II’s higher rejection rates for the main effects.

The claim that results obtained with SS III are unbiased [1] does not seem to be warranted. We found lower effect estimates and consequently, lower rejection rates of H0 of both main effects. As this easily amounts to a 5% lower rejection rate of H0, the estimates can be considered as conservative.

A most relevant question is under what conditions interaction can be re-estimated correctly and under what conditions it cannot. As the answer is that this is dependent on the way the effects are realized, it is impossible to answer this question completely. As we rarely have precise information on how the effects are realized in empirical research, it is only possible to formulate assumptions about it. In simulations, we can control the construction of the effects, and our results show that when both control and treatment groups contribute equally to the group differences, the interaction can be re-estimated correctly. However, if we generate data under the assumption that only the treatment group show effects, the interaction is not re-estimated correctly. Regretfully, the latter assumption seems most reasonable for factorial experiments with no-treatment control groups. Other assumptions are also plausible, for instance that both a standard treatment and a new experimental treatment both have different positive contributions to the outcomes. Clearly, more research on this point is desirable.

One might argue that the real choice is between accepting both possible over- and underestimation (SS II), and accepting only underestimation (SS III). The first would be a reason for choosing SS II in the presence of an interaction, as has been argued by Langsrud and Macnaugton [5,6]. In the presence of an interaction effect, SS II can easily result in a rejection rate of H0 of more than 10% higher than the rejection rates of balanced datasets drawn from the same population, which can be seen as a substantial increase of the Type I Error. Furthermore, when the estimation of the unique interaction effect is unsatisfactory, this can hardly be considered as an improvement of the analysis. In our view, the results provide no argument for extension of the use of SS II, other than when the presence of an interaction can be excluded with certainty.

The translation from the simulations in this study towards individual cases of unbalanced 2 x 2 datasets is complex. It is evident that if no interaction effect can be present, the use of SS II remains the best choice. As we have shown, certainty about the presence or absence of an interaction effect is difficult to acquire. SS III produces conservative estimates and the chances of overestimation of the main effects are practically zero while the results of SS II vary widely. If no interaction is present, our results also show that the advantages of SS II over SS III are relatively small. It is perhaps best to apply SS III in the case of unbalanced datasets whenever an interaction is conceivable. In our view, it is not very relevant whether this interaction is statistically significant or not.

## Supporting Information

S1 File “S1 ANOVA—Venn Diagrams.r”. This file is for calculating and displaying 2 X 2 ANOVA Venn-diagrams.
(R) Click here for additional data file.

S2 File “S2 multichoose.r”. This file is for generating the set of unbalanced designs.
(R) Click here for additional data file.

S3 File “S3 simulate unbalanced01.R”. This file is for simulating unbalanced data with zero contribution of the control groups.
(R) Click here for additional data file.

S4 File “S4 simulate unbalanced-11.R”. This file is for simulating unbalanced data with equal contributions to the effect of both control and treatment groups.
(R) Click here for additional data file.

S5 File “S5 create unbalanced 2x2.r”. Create the metafiles of the simulated datasets for further analysis.
(R) Click here for additional data file.

S6 File "S6 simdata01.txt". The first resulting dataset.
(TXT)

S7 File "S7 simdata-11.txt". The second resulting dataset.
(TXT)

S8 File “S8 analyze unbalanced.r”. File for analyzing the simulated datasets and generating tables.
(R) Click here for additional data file.
